# A 'spicy' encephalopathy: synthetic cannabinoids as cause of encephalopathy and seizure

**DOI:** 10.1186/s13054-014-0553-6

**Published:** 2014-10-20

**Authors:** Irene K Louh, William D Freeman

**Affiliations:** Department of Internal Medicine at Mayo Clinic, Jacksonville, FL 32224 USA; Division of Pulmonary, Allergy, and Critical Care Medicine, Columbia University, New York, NY 10032 USA; Department of Neurology at Mayo Clinic, Jacksonville, FL 32224 USA; Department of Critical Care at Mayo Clinic, Jacksonville, FL 32224 USA; Department of Neurosurgery at Mayo Clinic, Jacksonville, FL 32224 USA

Synthetic cannabinoids, often sold under labels such as 'spice', are a popular product sold in incense shops and on the internet. When inhaled, consumers often report experiences similar to marijuana use, thus making synthetic cannabinoids a popular street substitute for marijuana. With increasing use, the number of patients presenting to emergency departments due to the toxic effects of these products has increased. While many reported side effects, including anxiety, agitation, tachycardia, and hypertension [1,2], are transient and relatively mild, reports of more severe consequences, including psychosis and seizures, are increasing [2,3] with ICU admissions.

**Figure 1:**
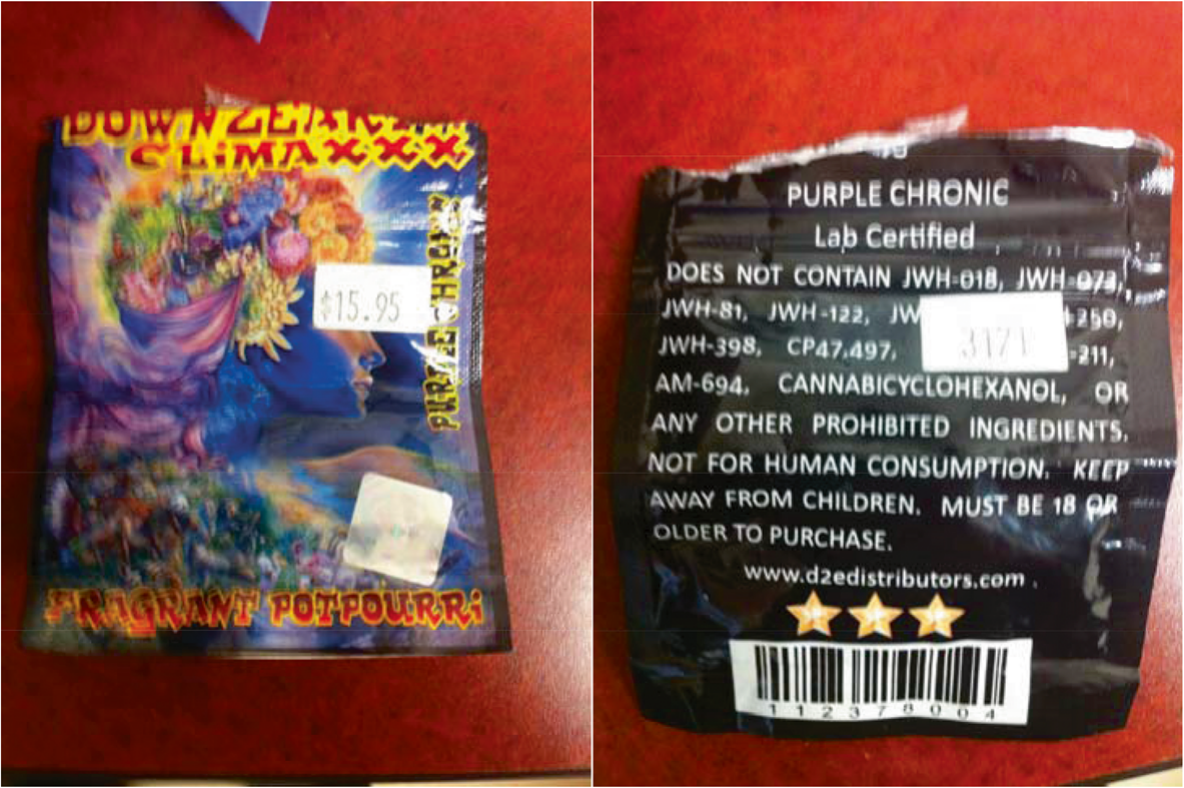
**Spice packaging.** Image of 'spice' incense packaging. Note the description of 'fragrant potpourri' and 'not for human consumption' on packaging.

Spice is often sold as 'incense' in tobacco shops and because of such is not under federal regulations for human consumption (Figure 1 shows the disclaimer on the packaging that it is not to be used for human consumption). According to data from the National Poison Data System, 3.8% of calls regarding intoxication from synthetic cannabinoids in 2010 reported seizures [3], and in 2013, there were 2,613 calls to poison control centers for synthetic cannabinoid exposure [4]. We find that spice intoxication is challenging to diagnose clinically because the features mimic serotonin syndrome. For example, we recently saw a case of 'spice encephalopathy' that presented as a first-onset seizure but had features of serotonin syndrome (myoclonus, dilated pupils). However, serotonin syndrome and spice intoxication have signs and symptoms that may overlap with other toxic drug ingestion states. Further, when reviewing the literature on ‘spice', we found eight other discrete cases demonstrating seizure post-'spice' inhalation. In all reported cases, toxicology screens for cannabis are negative, such as ours was. This is, in fact, to be expected with synthetic cannabinoid intoxication and part of the diagnostic dilemma. All reported cases of spice intoxication are similar in that seizure may manifest within a few hours after smoking the synthetic cannabinoid. In addition, some patients require intubation and mechanical ventilation requiring ICU admission, but most recover quickly. Our patient’s case was admitted to the ICU after a bag of synthetic spice was noticed along with a partially smoked ‘spice cigarette’, which confirmed the diagnosis. As signs/symptoms can be similar to serotonin syndrome, it is important to review carefully the medical history and medications or historians who find such patients since they may not be able to provide a history themselves. A high degree of clinical suspicion of this drug is required, and discovery of the smoking material (Figure 1) as in our case can greatly aid in the clinical diagnosis until more accurate laboratory methods can confirm this toxic substance in toxicology tests.

'Spice' or synthetic cannabinoid-induced toxicity is an emerging etiology of new-onset seizure and does not appear on conventional drug screens. We feel it is important for critical care providers to be aware of this product in order to recognize and appropriately treat this toxicity with supportive management until symptoms resolve. Obtaining additional history about smoking these substances can be helpful in making a clinical diagnosis until more widespread laboratory testing becomes available.
